# Effect of the 2015 earthquake on pediatric inpatient pattern at a tertiary care hospital in Nepal

**DOI:** 10.1186/s12887-018-1008-z

**Published:** 2018-02-05

**Authors:** Bishnu Rath Giri, Ram Hari Chapagain, Samana Sharma, Sandeep Shrestha, Sunita Ghimire, P. Ravi Shankar

**Affiliations:** 1 Department of Pediatrics, National Academy of Medical Sciences, Kanti Children’s Hospital, KMPC-3, Maharajgunj, Kathmandu, Postal Code: 44606 Nepal; 2 American International Medical University, Gros Islet, Saint Lucia

**Keywords:** Children, Critical care, Earthquake, Effect, Glomerulonephritis, Health, Inpatient, Nepal, Pediatric

## Abstract

**Background:**

Earthquakes impact child health in many ways. Diseases occurring immediately following an earthquake have been studied in field based hospitals but studies on the inpatient disease pattern among children without trauma in a permanent hospital setup is lacking.

**Methods:**

We examined the diagnoses of all children without trauma, admitted to Kanti Children’s Hospital, Kathmandu for fifteen-week duration (from 4th week to end of the 18th week) following the 7.8 magnitude Nepal earthquake on 25th April 2015. The admitted children were grouped based on direct effect of earthquake on their family (house damaged or family member injured or dead) and on whether their place of residence was located in an earthquake affected district. Most common diagnoses were identified and their distribution between the aforementioned groups analyzed to examine differences, if any, in disease occurrence or presentation. The fifteen weeks study duration was divided into three parts of five weeks each, to study trends in illness presentation. Variables were compared among various groups using appropriate statistical tests (*p* < 0.05).

**Results:**

A total of 1057 patients were admitted. The proportion of patients requiring admission for pneumonia, acute gastroenteritis and acute or poststreptococcal glomerulonephritis (AGN/PSGN) was significantly higher among children belonging to earthquake affected districts. Proportion of patients with any infective condition was also significantly higher in this group. Acute gastroenteritis and any infective condition were significantly higher among children from substantially affected families.

The proportion of AGN/PSGN among admitted patients increased in successive time categories among patients from affected districts and from substantially affected families. Urinary Tract Infection, bronchiolitis, tuberculosis, pleural effusion, protein energy malnutrition/failure to thrive, nephrotic syndrome, meningitis/meningoencephalitis, epilepsy or seizure disorders, leukemia/malignancies, enteric fever, infective hepatitis and congenital heart disease were not significantly different among children from affected and not affected districts or between substantially affected and not affected families. Patients from substantially affected families were admitted to semi-intensive care ward or ICU in significantly higher proportions (12.6% vs 7.8%, *p* = 0.014).

**Conclusion:**

Children seeking care for certain diseases were more likely to be from earthquake affected families and districts. Those from affected families required critical care more often.

**Electronic supplementary material:**

The online version of this article (10.1186/s12887-018-1008-z) contains supplementary material, which is available to authorized users.

## Background

Nepal is a developing country situated in South Asia with an area of 1,47,181 km^2^ and an estimated population of 28.4 million. Children below 14 years of age comprise 34.6% of the population [1]. Per capita gross national income is US dollars ($) 743 in absolute terms ($2500 in terms of purchasing power parity (PPP) [2]. The difficult terrain limits access to healthcare facilities. Healthcare delivery system comprises of primary and preventive care provided through health posts and primary health care centers, secondary level of care provided at district and zonal hospitals and tertiary level care provided through hospitals in major cities. According to the Nepal multiple indicator cluster survey 2014 the neonatal mortality rate was 23 and the under-five year’s mortality was 38 per thousand live births. The same study revealed that 37.4% of the children below five years of age were stunted, 11.3% wasted and 30.1% were underweight [3].

Geologically, Nepal lies over the fault line of the Indian and Eurasian plates and is an earthquake prone zone. Geographically the northern part of the country consists of the Himalayas including Mount Everest and the lesser Himalayas whereas the southern part of the country consists of the plains of the Terai. For administrative purposes, the country has been divided into 75 districts. The epicenter of the Gorkha earthquake, that hit the region on 25th April 2015 with a magnitude of 7.8, was nearly in the center of Nepal, 77 Kilometers northwest of the capital city of Kathmandu. The earthquake and its aftershocks affected thirty-nine districts. Fourteen districts, including the capital Kathmandu, were classified as the *most affected*. The earthquake caused 8856 deaths and injured 22,309 people. Sindhupalchowk was the worst hit district with 3532 reported deaths and 100% of houses damaged followed by Kathmandu where the death toll stood at 1226 and 7952 were injured [4]. For all the affected districts, Kathmandu is the major hub in terms of trade, transportation, administrative works and health care. Kanti Children’s Hospital, located in Kathmandu is the only government run tertiary care general hospital for children less than 14 years of age in the whole country and thus is an ideal site for studying disease and inpatient admission pattern among children during the post rescue phase of the earthquake.

Data from a study conducted at a pediatric field-based hospital following the Haiti earthquake found that while 93 % of inpatients on the ninth day after the quake were surgical, eight weeks later, 71 % of the inpatients had medical illnesses [5]. Hung et al. in their study in rural China following the Sichuan earthquake pointed out the possibility of exacerbation of pre-existing chronic illnesses in the community following disasters [6]. Most articles studying the effect of earthquake on children’s health had examined the immediate consequences and focused on trauma events [7–11]. Articles in the literature that specifically deal with medical illnesses among children during the recovery phase of an earthquake were scarce. We thus set out to conduct this study with the objectives of examining:

- Differences, if any, in inpatient admission pattern among children from earthquake affected families and unaffected families
- Differences, if any, in inpatient pattern among children from earthquake affected and unaffected districts.

## Methods

All patients admitted to the general pediatrics department, with non-traumatic or non-surgical illnesses, beginning from the fourth week (twenty-second day) till the end of the eighteenth week (one hundred and twenty-sixth day) after the earthquake on 25th April 2015 were included in the study (16th May to 28th August 2015). Study approval and ethical clearance was obtained from the institutional review committee of Kanti Children’s Hospital and verbal consent was obtained from the guardian of the child prior to data collection. Data on demographics and effects of the earthquake on the child’s family was obtained by interviewing the patient’s guardian and clinical data was obtained from the patient’s case record.

Kanti Children’s hospital is a 320 bedded children’s tertiary care general hospital which gets referred patients below 14 years of age from all over the country and also caters to patients arriving directly without referral. The services include out-patient, emergency, general pediatrics, pediatric surgery, neonatal and pediatric intensive care, immunization and other preventive services. It has a free ward with 50 beds dedicated to poor patients. Some of the patients in this ward get the necessary investigations and certain medicines free from the hospital supply but medicines mostly have to be purchased from the hospital or outside pharmacies by the patients’ families. Critically ill patients may be directly transferred to intensive care units from emergency or observation wards. For patients in the general ward there is no designated residential facility for guardians other than the one staying with the patient in the ward.

Data from individual patients was collected using a proforma (Additional file 1: Proforma_EEPIP) designed for this purpose, which included demographic details, the presence of and degree of damage to the house and its consequences including displacement to temporary shelter, death or injury to parents or other family members, diagnosis, duration of hospital stay and requirement for critical care (transfer to Intermediate dependency or Intensive Care Units).

For analysis, the effect of the earthquake on the patient and family was studied using two frameworks. These frameworks were developed by the authors following discussions and a review of the scientific literature. The first classification framework was the impact of earthquake on the family and/or house. The impact was divided into two categories: first, *substantially affected* category - defined as at least one parent injured or dead or the family displaced to temporary shelter due to complete damage to the house and second, *no or minor effect* category - defined as no physical harm to parents and either no damage to house or house damaged but the family continued to live in the same house or some other house but not in temporary shelter. The second classification framework was whether or not the place of residence of the patient was located in *the affected districts* as per the government’s classification [4]. The ‘most affected districts were classified as *‘affected districts’,* all other districts including less affected ones were classified as *‘non-affected districts’*. The groups within these classification frameworks were examined for any difference in the occurrence of disease conditions or disease presentation. To examine the change in disease pattern with time, the total fifteen weeks duration of the study was divided into three parts of five weeks each.

Data analysis was performed using IBM statistical package for social sciences (SPSS) version 22 for Windows. Demographic and clinical variables were compared separately between the groups within each classification framework. Continuous variables were compared using student’s *t*-test, and categorical variables were compared using chi square test. A *p*-value < 0.05 was considered statistically significant.

## Results

A total of 1057 children were admitted in general pediatrics department during the study period. Among these 7 children had at least one of their parents dead while 23 children (2.2%) had at least one of their parents injured due to the earthquake. Because of complete damage to their house, 326 children (30.8%) were living in temporary shelters; 327 (30.9%) were in the ‘substantially affected’ group and the remaining 730 (69.1%) were in the ‘no or minor effect’ group. Based on district of residence, 586 (55.5%) belonged to ‘affected districts’ and 470 (45.5%) belonged to non-affected districts. On combining the variables, district of residence and the effect on the family, 53.1% of children from ‘affected districts’ were in the ‘substantially affected’ group, while the figure was 3.2% for children from non-affected districts.

Table 1 show the demographic and baseline characteristics of the patients in different groups. Mean walking distance from the home to the nearest bus stop and mean duration of symptoms were significantly higher among patients from non-affected districts. Significantly higher proportion of patients from affected districts and from substantially affected families were admitted to the free ward. Significantly higher proportion of patients from substantially affected families required some form of critical care compared to families with minor or no effect (12.54 vs 7.51%, *p* = 0.014).

**Table 1:** Demographic and baseline characteristics of admitted children (*n* = 1057)

	Based on district of residence*			Based on direct effects on family/house		
Characteristics	Non-affected districts (*n* = 470)	Affected districts (*n* = 586)	*p*-value	Not substantially affected (*n* = 730)	Substantially affected (*n* = 327)	*p*-value
Mean age in months (sd)	48.4 (49.53)	52.27 (50.39)	0.212	48.38 (49.19)	55.45 (51.53)	0.034
Gender (percentage of male patients)	68.9	57.8	< 0.001	66.71	54.13	< 0.001
Mean weight in kg (sd)	14.10 (9.73)	15.54 (10.54)	0.022	14.37 (9.99)	16.09 (10.58)	0.073
Mean walking distance in minutes from nearest bus stop (sd)	100.76 (435.08)	43.05 (249.45)	0.007	74.13 (353.31)	56.84 (328.48)	0.453
Mean duration of symptoms in days (sd) *n* = 990**	10.64 (12.38) (*n* = 434)	7.40 (9.44) (*n* = 556)	< 0.001	9.13 (11.99) (*n* = 685)	8.13 (10.79) (*n* = 305)	0.186
Admissions in free ward out of all general ward admissions (%) *n* = 963***	51.7 (*n* = 532)	60 (*n* = 431)	0.011	51.56 (*n* = 678)	67.5 (*n* = 286)	< 0.001
Requirement for critical care (percent)	9.36	9.22	0.935	7.81	12.54	0.014

*one patient’s district of residence data was missing

**patients with onset of symptoms prior to the earthquake were excluded

***remaining patients were directly admitted to wards with some level of critical care

Pneumonia (isolated or with predisposing condition) was the most common diagnosis (22.3%) and was in significantly higher proportion among patients from affected districts compared to those from non-affected districts (26.6 vs 17%). However, the difference was not significant among patients from substantially affected and not affected families [Table 2]. Occurrence of acute or poststreptococcal glomerulonephritis (AGN/PSGN), acute gastroenteritis, respiratory infections (including pneumonia) and other infective conditions was significantly higher whereas that of sepsis, fever with no identified focus and anemia were significantly lower among patients from affected districts compared to non-affected districts. On comparison between patients from substantially affected families and not affected ones, acute gastroenteritis and infective conditions (all cases with proven or presumed infectious etiology) were in significantly higher proportion whereas fever with no identified focus and anemia were in significantly lower proportion in the former group.

**Table 2:** Occurrence of diseases (percentage) among admitted children (*n* = 1057)

	Based on district of residence*			Based on direct effects on family/house		
Disease/condition	Not affected districts (*n* = 470)	Affected districts (*n* = 586)	*p*-value	Not substantially affected (*n* = 730)	Substantially affected (*n* = 327)	*p*-value
Pneumonia	17	26.6	< 0.001	20.8	25.7	0.079
Sepsis	18.1	13.3	0.033	16.4	13.1	0.171
Fever without a focus	4.89	1.71	0.003	3.97	1.22	0.018
AGN/PSGN	1.1	4.3	0.002	2.2	4.3	0.059
A/c Gastroenteritis	0.9	2.6	0.038	1	3.7	0.002
Anemia	4	0.9	0.001	3.0	0.6	0.015
Infective conditions*	55.7	67.9	< 0.001	61.3	64.6	0.047
Respiratory Infections	23.0	33.8	< 0.001	28.1	30.9	0.353
GI Infections	7.2	9.7	0.151	7.5	11	0.063

*compared after cases were categorized as infective, non-infective and fever without a focus

Urinary tract Infection, bronchiolitis, tuberculosis (pulmonary and extrapulmonary), pleural effusion, protein energy malnutrition/failure to thrive, nephrotic syndrome, meningitis or meningoencephalitis, epilepsy or seizure disorders (febrile seizure not included), leukemia/malignancies, enteric fever, infective hepatitis and congenital heart disease (with or without complications) were not significantly different either between children from the affected and not affected districts or between substantially affected and not substantially affected families.

The proportion of patients with AGN/PSGN increased in each successive five week category. The rise was statistically significant among patients from affected districts (compared to non-affected districts) as well as those from substantially affected families (compared to families not substantially affected) [Table 3].

**Table 3:** Percentage of acute glomerulonephritis/poststreptococcal glomerulonephritis over successive five-week intervals of the study

		Percentage of AGN/PSGN			*P* value *(Chi-Square test)*
		4–8 weeks	9–13 weeks	14–18 weeks	
Belonging to affected district	Yes (*n* = 586)	1.4	2.4	6.2	0.035
	No (*n* = 470)	0.0	0.9	1.7	0.337
Direct effect of earthquake	Substantial (*n* = 327)	0.9	3.0	7.1	0.044
	Not substantial (*n* = 730)	0.7	1.2	3.2	0.11
Total (*n* = 1057)		0.8	1.7	4.3	0.009

Chi square test for trend was used for percentage comparisons across the different time intervals

## Discussion

We found infectious diseases to be significantly higher among children from affected districts and affected families. Pneumonia was the most common diagnosis, seen in higher proportion among children from affected districts. Pneumonia and respiratory infections were also common in other studies reported in the literature. Following the 2009 Sumatra quake, the Singapore armed forces medical team recorded that up to 47% of the patients in their mobile clinic had pneumonia or upper respiratory tract infection (URTI) [12]. Among outpatients in a permanent primary care facility in Kaghan, Pakistan following the 2005 earthquake, viral URTI was found to be the most common illness accounting for 23 % of all cases [13]. In a survey one year following the Nepal earthquake, children from affected districts reported suffering from respiratory infections and diarrhea more often compared to before the earthquake [14].

Diarrheal diseases accounted for more than 40% of deaths in camps during the acute phase of an emergency, with over 80% of these deaths occurring among children less than two years old [15]. In our study, acute gastroenteritis requiring admission was seen in higher proportion among children from affected districts as well as affected families, but the total number of cases was less. This is because most cases of diarrheal illnesses were treated in the observation ward and discharged after stabilization without being admitted. Another factor to be considered is that patients from non-affected districts, which are more distant, may not travel to Kathmandu for self-limiting or short-term illnesses. This factor might have influenced the frequency of other diseases in our analysis of patients from affected and non-affected districts.

Risk of epidemics of infectious diseases following earthquakes has been mentioned with suggestions for investing in sanitation, hygiene and vaccination [16]. Outbreaks of diarrhea, cholera, hepatitis E, ARI, influenza, measles, meningitis and tetanus have been reported following major earthquakes during the last decade [17].

The World Health Organization (WHO) estimated that about 2.8 million people were displaced following the Nepal earthquake and warned of possible outbreaks of water-borne diseases, vector-borne diseases and acute respiratory infections. The organization also warned of worsening of child nutrition status [18]. Fortunately, there has been no major disease outbreak reported in the earthquake affected districts in the months following the earthquake. Our study also did not find a significant rise in many of the diseases reported to have been in epidemic proportions in other disasters or anticipated to be a possible epidemic in this disaster such as measles, malnutrition, cholera, enteric fever etc. The present study was a hospital-based one and population-based assessments would be needed to study the incidence of illness following the earthquake.

Many public health measures had been undertaken at the community level by different agencies including government primary health institutions, but very few of these have been documented and published. The published activities range from distribution of sanitation hygiene kits to administration of 18,000 doses of cholera vaccine [19, 20]. According to a report by the United National International Children’s Emergency Fund (UNICEF), in some areas children were immunized against measles, polio and rubella; they were screened for acute malnutrition and 82,000 families received family hygiene kits [21].

Children from affected families may have more severe illness at presentation possibly because the impact of the earthquake on the family/care givers and/or diverted attention of the family toward the task of finding shelter and basic necessities. Burnweit et al. mentioned in their article that by the eighth week following the earthquake, one third of the admissions in their field based pediatric hospital required critical care [5]. Dulski et al. found that among non-injured patients following an earthquake, admission to neonatal or pediatric intensive care unit (NICU/PICU) is a significant predictor of death [22]. Sudden increase in patients requiring critical care in resource limited settings, necessitates allocation of resources according to distributive-justice principle (community centered approach) rather than autonomy based principle (patient centered approach) where subjective considerations like family wishes and values are replaced with objective considerations like prognostic criteria as pointed out by Ytzhak et al. [23]. This approach aims to provide treatment to maximum number of patients with expected good outcome by use of minimum resources for relatively shorter period of time.

One interesting observation in our study is the sequential rise in the proportion of acute glomerulonephritis in successive weeks following the earthquake. The most plausible explanation for this temporal rise in AGN cases would be that children were more likely to have streptococcal infection, most likely that of the throat, following earthquakes and similar disasters, this infection rises in incidence with passage of time and AGN develops in those children infected with nephritogenic strains. We did not find any similar study with this finding, and this could be examined in future studies.

### Strengths and limitations

Our study presents data from a fixed tertiary care children’s hospital located in the center of the earthquake affected region in the capital of Nepal. Data on medical illnesses during the recovery phase of an earthquake is also presented. Such data is limited in the literature. We considered the diagnosis at admission for analysis due to logistic and resource considerations, but the final diagnosis would have been a better variable.

Another weakness of the study is due to geographic proximity of Kathmandu to the affected districts. The non-affected districts were located at a greater distance from Kathmandu thus children with non-serious illnesses and illnesses of short duration such as gastroenteritis were less likely to travel to Kathmandu for treatment. This could have affected the comparison of disease pattern among affected and non-affected districts. The less affected or not affected districts which lie farther from Kathmandu are relatively remote and the road network is less developed. Thus the place of residence for patients from non-affected districts may be farther from the nearest bus stop compared to those from affected districts. Patients from non-affected districts presented significantly later than those from affected districts after onset of disease symptoms. Geographical proximity to Kathmandu and Kanti Children’s Hospital for patients from affected districts might have contributed to this difference.

Our study deals with admitted patients in a tertiary care hospital, many children affected by diseases might have received primary health care in the community itself. However, some children may not have been able to access care at all. Thus our study does not reveal the total post-earthquake disease epidemiology in the community.

## Conclusion

The effect of an earthquake in terms of health hazards is not limited to direct trauma or the risk of epidemics, but can affect the whole community for a prolonged period. The pattern of certain diseases among children from substantially affected families was different from those not substantially affected and the former group of children required critical care more often. Certain diseases were significantly higher among children from affected districts. The proportion of AGN/PSGN among admitted patients from affected districts and from substantially affected families increased during the three successive time periods. The limitations of the study mentioned previously could limit the generalizability of the study conclusions.

We recommend further studies examining the effect of earthquake on medical illnesses among both admitted and non-admitted children, for an extended duration following an earthquake. Based on our results we recommend improved planning and preparedness for dealing with medical illnesses that may arise during the recovery phase of disasters especially among children.

## Additional file

Additional file 1: Proforma: Effect of Earthquake on Pediatric Inpatient Pattern. Data collection instrument. (DOCX 12 kb)

## Abbreviations

AGN: Acute Glomerulonephritis
FTT: Failure to Thrive
ICU: Intensive Care Unit
IRC: Institutional Review Committee
NHRC: Nepal Health Research Council
NICU: Neonatal Intensive Care Unit
PEM: Protein Energy Malnutrition
PICU: Pediatric Intensive Care Unit
PPP: Purchasing Power Parity
PSGN: Post Streptococcal Glomerulonephritis
UNICEF: United Nations International Children’s Emergency Fund
URTI: Upper Respiratory Tract Infection
WHO: World Health Organization

## References

[1] Population Monograph of Nepal 2014. In. Central Bureau of Statistics Kathmandu, Nepal; 2014. http://cbs.gov.np/population/populationmonographnepa_2014. Accessed 29 Jan 2017.

[2] World Development Indicators: Country Nepal. In. World Bank Group; 2015. https://data.worldbank.org/country/nepal. Accessed 29 Jan 2017.

[3] Nepal Multiple Indicator Cluster Survey 2014, Final report. In: central Bureau of Statistics and UNICEF Nepal; 2015. http://unicef.org.np/uploads/files/597341286609672028-final-report-nmics-2014-english.pdf. Accessed 29 Jan 2017.

[4] Key Findings (2015). Nepal earthquake 2015 post disaster need assessment.

[5] Burnweit C, Stylianos S (2011). Disaster response in a pediatric field hospital: lessons learned in Haiti. J Pediatr Surg.

[6] Hung KK, Lam EC, Chan EY, Graham CA (2013). Disease pattern and chronic illness in rural China: the Hong Kong red cross basic health clinic after 2008 Sichuan earthquake. Emerg Med Australas.

[7] Zhao J, Shi Y, Hu Z, Li H (2011). Sichuan earthquake and emergency relief care for children: report from the firstly arrived pediatricians in the epicenter zone. Pediatr Emerg Care.

[8] Zhang TC (2013). Types of pediatric trauma in earthquake and key points of treatment. Zhongguo Dang Dai Er Ke Za Zhi.

[9] Ding H, Fan H, Lv Q, Liu Z, Zhang Y, Hou S (2015). Analyses of the disease Spectrum of children after the Lushan earthquake. Pediatr Emerg Care.

[10] Jacquet GA, Hansoti B, Vu A, Bayram JD. Earthquake-related injuries in the pediatric population: a systematic review. PLoS Curr Disasters. 2013. Edition 1. 10.1371/currents.dis.6d3efba2712560727c0a551f4febac16. 10.1371/currents.dis.6d3efba2712560727c0a551f4febac16 PMC3992814 24761308

[11] Anand KJ, Eubanks JW, Kelly DM, Meier JW, Saltzman JA, Crisler SC (2010). Pediatric patients seen in Port-au-Prince, Haiti. Clin Pediatr (Phila).

[12] Tan CM, Lee VJ, Chang GH, Ang HX, Seet B (2012). Medical response to the 2009 Sumatra earthquake: health needs in the post-disaster period. Singpore Med J.

[13] Shah N, Abro MA, Abro MA, Khan A, Anwar F, Akhtar H (2010). Disease pattern in earthquake affected areas of Pakistan: data from Kaghan valley. J Ayub Med Coll Abbottabad.

[14] Fievet V, Singh K, Davis A (2016). Children's Voices, Children's rights: One year after the Nepal Earthquake. Plan international, Save the Children, UNICEF, World Vision International.

[15] Connolly MA, Gayer M, Ryan MJ, Salama P, Spiegel P, Heymann DL (2004). Communicable diseases in complex emergencies: impact and challenges. Lancet.

[16] Basnyat B, Tabin C, Nutt C, Farmer P (2015). Post-earthquake Nepal: the way forward. Lancet Glob Health.

[17] Kouadio IK, Aljunid S, Kamigaki T, Hammad K, Oshitani H (2012). Infectious diseases following natural disasters: prevention and control measures. Expert Rev Anti-Infect Ther.

[18] World Health Organization (2015). Nepal earthquake 2015 country update and funding request.

[19] Newman MS.The epidemic that wasn't. https://nextcity.org/daily/entry/nepal-earthquake-cholera-epidemic-solutions. Accessed 2 Feb 2017.

[20] Save The Children.Save the Children's Earthquake Response in Nepal: A Special One year Progress Report.Save the Children: Connecticut; 2016. http://www.savethechildren.org/atf/cf/%7B9def2ebe-10ae-432c-9bd0-df91d2eba74a%7D/SAVE%20THE%20CHILDREN%20NEPAL%20ONE-YEAR%20REPORT%20APRIL%202016.PDF. Accessed 3 Feb 2017.

[21] United Nations Children's Fund.UNICEF Annual Report 2015, https://www.unicef.org/publications/files/UNICEF_Annual_Report_2015_En.pdf. Accessed 3 Feb 2017.

[22] Dulski TM, Basavaraju SV, Hotz GA, Xu L, Selent MU, DeGennaro VA (2011). Factors associated with inpatient mortality in a field hospital following the Haiti earthquake, January-may 2010. Am J Disaster Med.

[23] Ytzhak A, Sagi R, Bader T, Assa A, Farfel A, Merin O (2012). Pediatric ventilation in a disaster: clinical and ethical decision making. Crit Care Med.

