# Supplementary material for: Effect of the 2015 earthquake on pediatric inpatient pattern at a tertiary care hospital in Nepal
Source: BMC Pediatr. 2018 Feb 5;18:28. doi: 10.1186/s12887-018-1008-z (PMC5800012; doi:10.1186/s12887-018-1008-z)
Supplement: Additional file 1: — Proforma: Effect of Earthquake on Pediatric Inpatient Pattern. Data collection instrument. (DOCX 12 kb) [file 12887_2018_1008_MOESM1_ESM.docx]

**Proforma: Effect of Earthquake on Pediatric Inpatient Pattern**

SN …………………

Name: Date of Admission in BS :

Age (months): Gender: Weight: DoA in AD :

Ward (medical/paying/cabin/intermediate/ICU) Bed No. :

Diagnosis at admission:

Chief complaints at admission (with duration):

Address (district and VDC):

Location of house (walking distance from nearest highway/buspark):

Contact number:

**Effects of earthquake:**

***Human loss:***

*Death:* One Parent

Both Parents

Others in family ……

*Injury:* One parent

Both Parents

Others in family ……

***House damage:***

Complete, living in temporary shelter

Complete, living in some other house

Partial, living in same house

No damage, living in same house

Date of discharge (AD):
